# Supplementary material for: Satiety, TAX-4, and OSM-9 tune the attraction of Caenorhabditis elegans nematodes to microbial fermentation products
Source: G3 (Bethesda). 2025 Oct 13;15(12):jkaf245. doi: 10.1093/g3journal/jkaf245 (PMC12693527; doi:10.1093/g3journal/jkaf245)
Supplement: jkaf245_Supplementary_Data [file jkaf245_supplementary_data.zip › Supplementary_Figure_Legends_G3-2025-406189.docx]

## Supplementary Figure Legends

**Supplementary Figure 1** | **Both fed and starved animals are indifferent to pH-buffered water**. Swarm plots (top) showing the location of individual worms (small dots) and the mean location derived from 16 replicates (large dots) for fed (**A**) or starved (**B**) wild-type worms. Vertical bars indicate the global 95% confidence interval; the gap between the bars shows the mean. The total number of animals is in parentheses. The mean difference (bottom) between each test condition and the reference (water on both sides) is plotted as a bootstrapped sampling distribution (<Δ location>). The central dot is the mean difference; the 95% confidence interval is shown by the ends of the vertical bar. Supplementary Table 4 contains the numerical values plotted in these graphs.

**Supplementary Figure 2** | **Responses of well-fed wild-type and mutant worms to biosynthetic precursors and fermentation products shown as swarm plots and differences of the mean location**. Swarm plots and bootstrapped differences of the mean location are color coded according to the tested compound, with gray representing the control (HEPES-buffered water), red representing the isoleucine degradation pathway (I1, I2, I3, and IAA), and blue representing the leucine degradation pathway (M1, M2, M3, and 2M1B). **A**. Wild-type animals, fed. *N* = 8 for all compounds. **B.** *osm-9* null animals, fed. *N* = 10 for HEPES-buffered water, I1, I3, IAA, M1, M2, and M3, *N* = 9 for I2, and *N* = 16 for 2M1B. **C.** *tax-4* null animals, fed. *N* = 9 for HEPES-buffered water, M2, and M3, *N* = 10 for I1, I2, I3, IAA, M1, and 2M1B. **D.** *osm-9; tax-4* animals, fed. *N* = 8 for all compounds. Small dots in each swarm plot (top) show the location of individual worms and large dots are the mean position of all the animals in a single replicate. Vertical bars indicate the global 95% confidence interval; the gap between the bars shows the mean. The total number of animals is in parentheses. The mean difference (bottom) between each test condition and the reference (HEPES on both sides) is plotted as a bootstrapped sampling distribution (<Δ location>). The central dot is the mean difference; the 95% confidence interval is shown by the ends of the vertical bar. Supplementary Table 5 contains the numerical values plotted in these graphs.

**Supplementary Figure 3** | **Responses of starved wild-type and mutant worms to biosynthetic precursors and fermentation products shown as swarm plots and differences of the mean location**. Swarm plots and bootstrapped differences of the mean location are color coded according to the tested compound, with gray representing the control (HEPES-buffered water), red representing the isoleucine degradation pathway (I1, I2, I3, and IAA), and blue representing the leucine degradation pathway (M1, M2, M3, and 2M1B). **A**. Wild-type animals, starved. *N* = 8 for all compounds. **B.** *osm-9* null animals, starved. *N* = 8 for all compounds. **C.** *tax-4* null animals, starved. *N* = 8 for all compounds. **D.** *osm-9; tax-4* animals, starved. *N* = 8 for all compounds. Small dots in each swarm plot (top) show the location of individual worms and large dots are the mean of all the animals in a single replicate. Vertical bars indicate the global 95% confidence interval; the gap between the bars shows the mean. The total number of animals is in parentheses. The mean difference (bottom) between each test condition and the reference (HEPES on both sides) is plotted as a bootstrapped sampling distribution (<Δ location>). The central dot is the mean difference; the 95% confidence interval is shown by the ends of the vertical bar. Supplementary Table 6 contains the numerical values plotted in these graphs.
