# Supplementary figures and images for: Satiety, TAX-4, and OSM-9 tune the attraction of Caenorhabditis elegans nematodes to microbial fermentation products
Source: G3 (Bethesda). 2025 Oct 13;15(12):jkaf245. doi: 10.1093/g3journal/jkaf245 (PMC12693527; doi:10.1093/g3journal/jkaf245)

**A**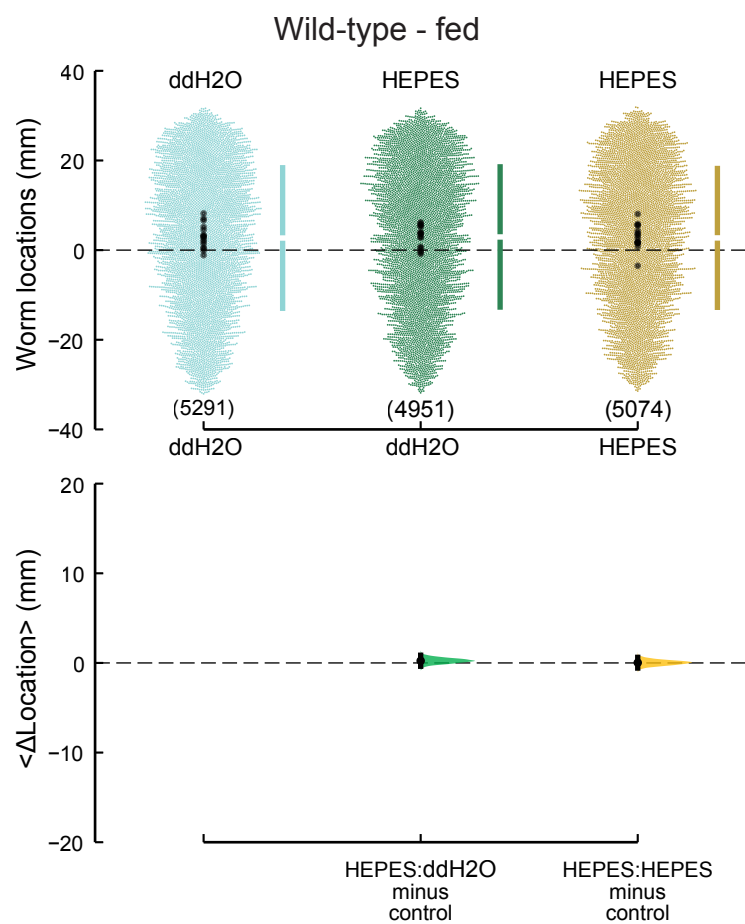**B**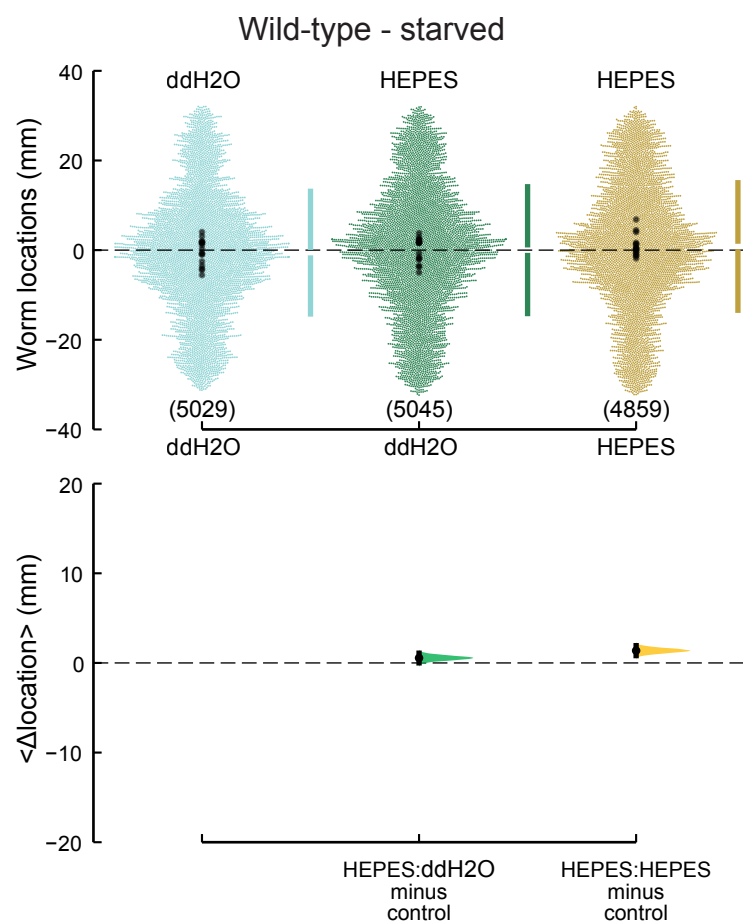

Supplement: jkaf245_Supplementary_Data [file jkaf245_supplementary_data.zip › Supplementary_Figure_1_G3-2025-406189.pdf]

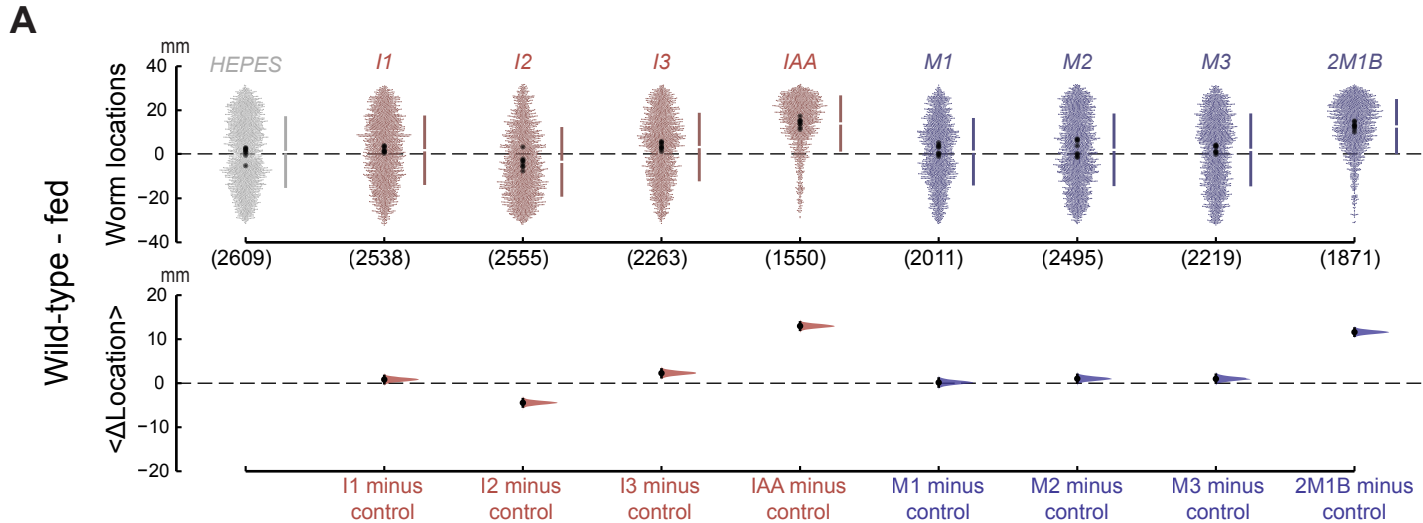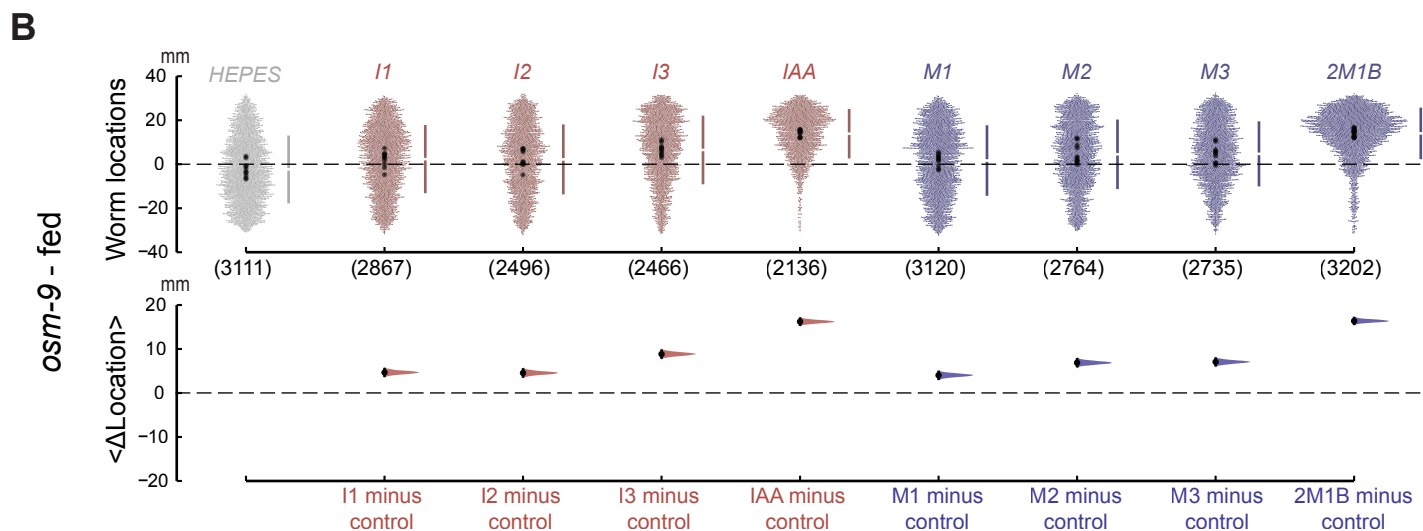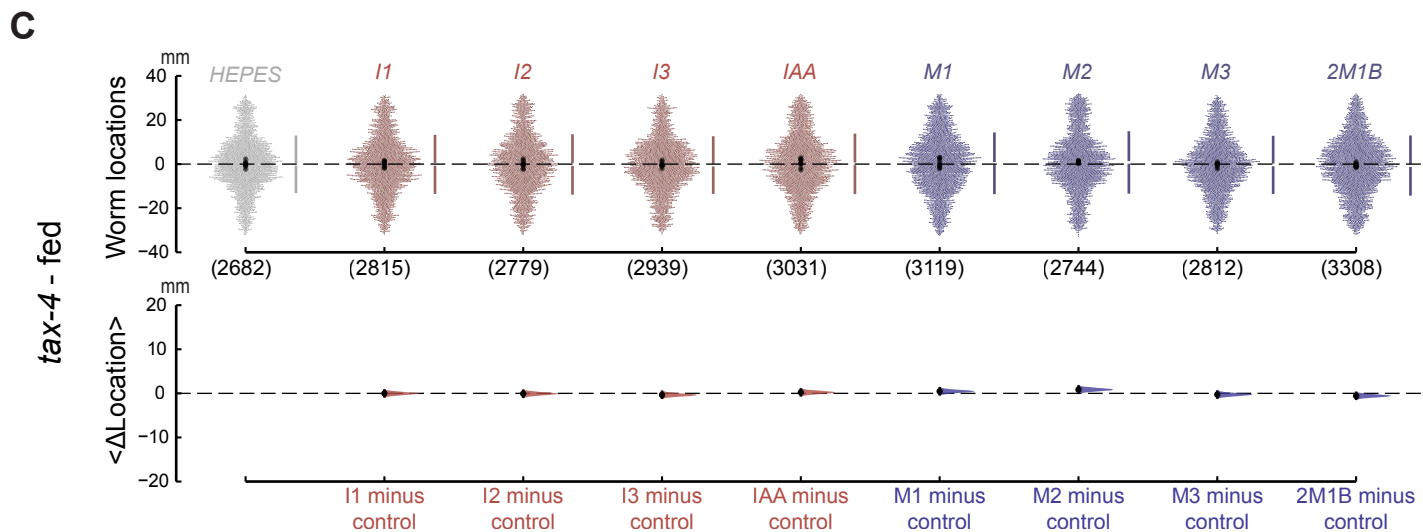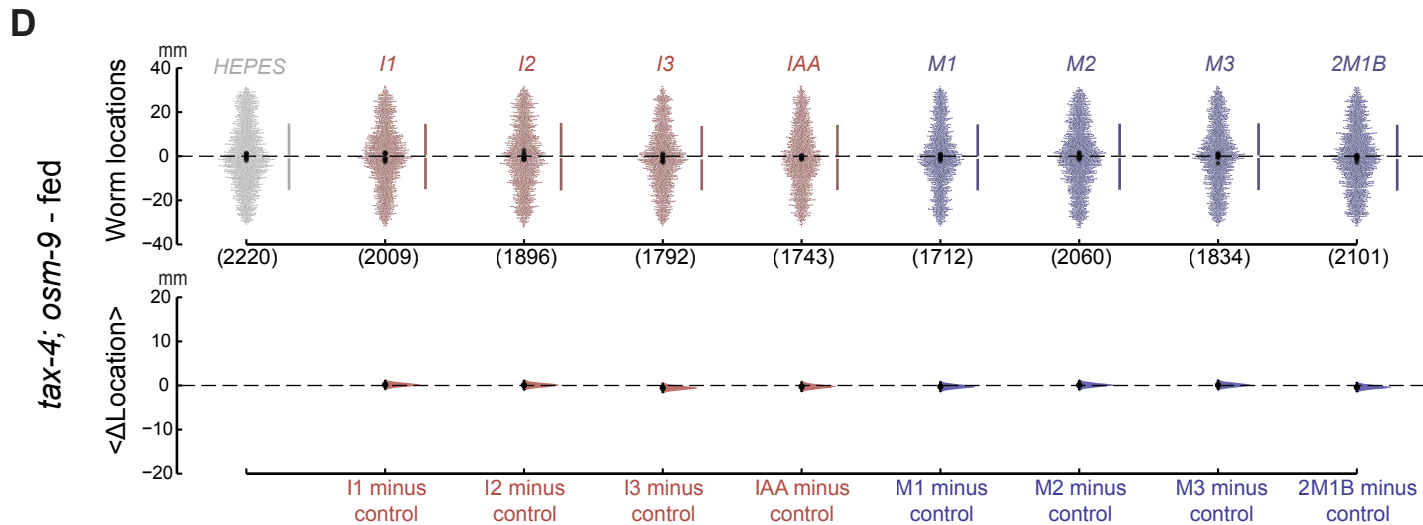

Supplement: jkaf245_Supplementary_Data [file jkaf245_supplementary_data.zip › Supplementary_Figure_2_G3-2025-406189.pdf]

**A**

Wild-type - starved

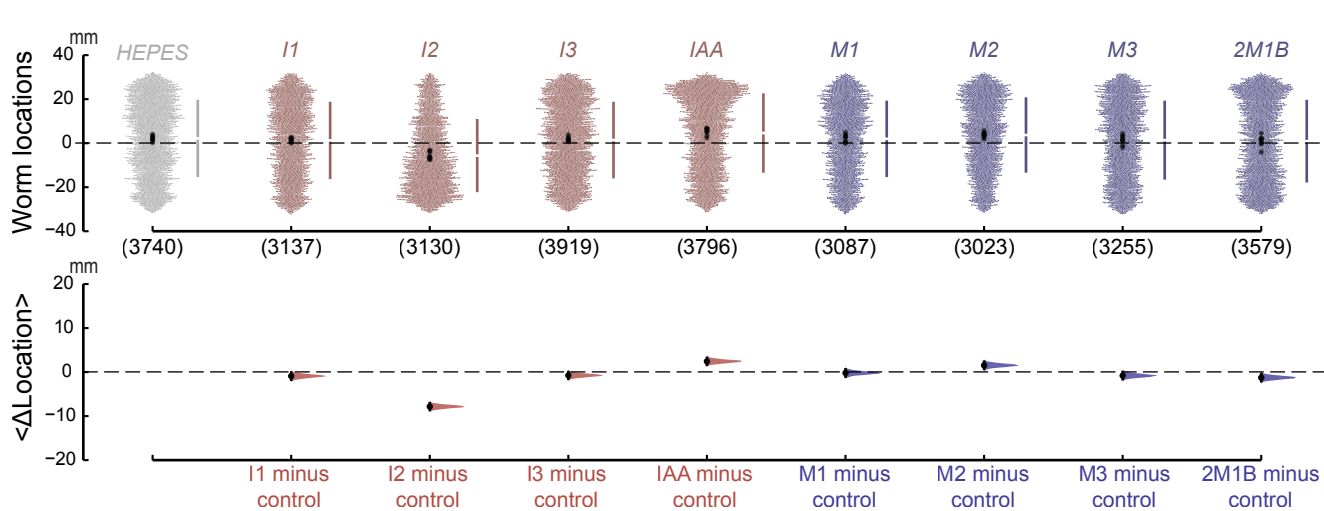**B***osm-9* - starved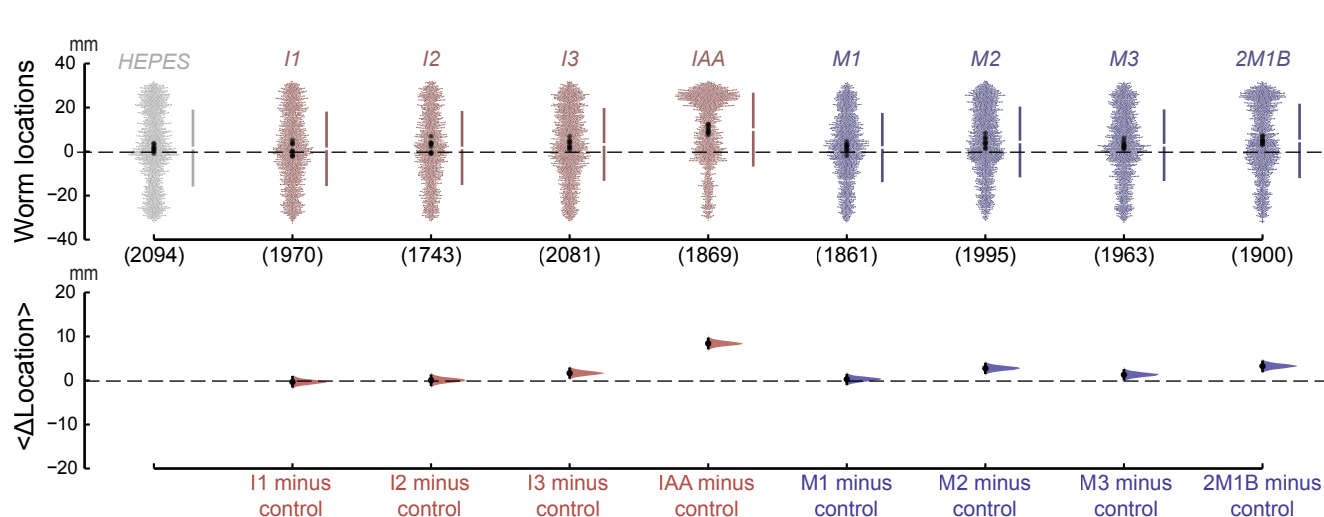**C***tax-4* - starved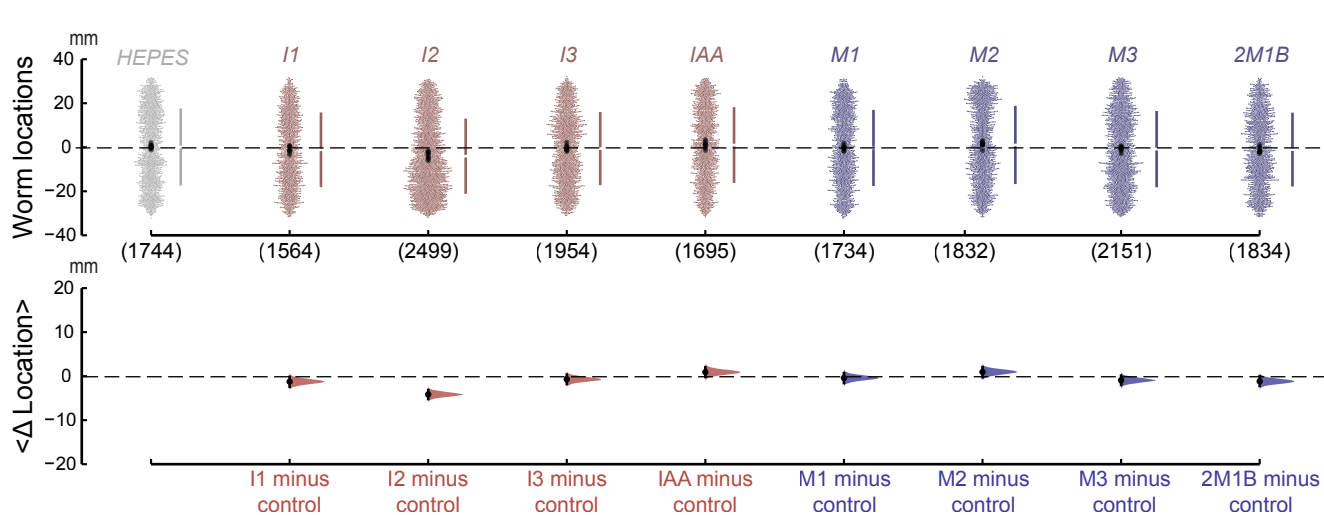**D***tax-4; osm-9* - starved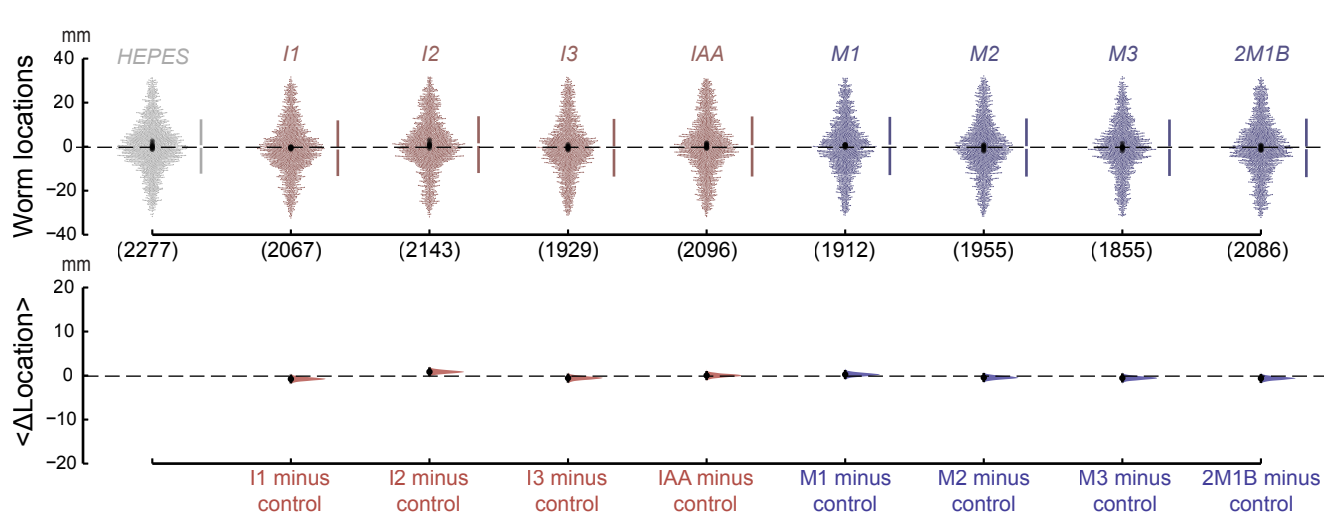

Supplement: jkaf245_Supplementary_Data [file jkaf245_supplementary_data.zip › Supplementary_Figure_3_G3-2025-406189.pdf]
